# Perceived Barriers to Serving on National Institutes of Health Scientific Review Groups Experienced by Black and African American Scientists

**DOI:** 10.1001/jamanetworkopen.2022.22085

**Published:** 2022-07-13

**Authors:** Eric K. Soule, Sabrina Ford, Robert L. Newton, Alisha Thomas, Thomas Eissenberg

**Affiliations:** 1 Department of Health Education and Promotion, East Carolina University, Greenville, North Carolina; 2 Center for the Study of Tobacco Products, Virginia Commonwealth University, Richmond; 3 Institute for Health Policy and the Department of Obstetrics, Gynecology, and Reproductive Biology, Michigan State University, East Lansing; 4 Population and Public Health, Pennington Biomedical Research Center, Louisiana State University, Baton Rouge; 5 Department of Psychology, Virginia Commonwealth University, Richmond

## Abstract

**Importance:**

African American and Black scientists are awarded disproportionately fewer National Institutes of Health (NIH) grants than White scientists. Increasing Black representation on NIH scientific review groups (SRGs) likely will contribute to increased equity in funding rates because research topics of Black and African American scientists’ submitted applications will be more highly valued; however, Black and African American scientists often perceive barriers that prevent them from serving on NIH SRGs.

**Objective:**

To examine perceived barriers that prevent Black and African American scientists from serving on NIH SRGs.

**Design, Setting, and Participants:**

This qualitative study used a mixed methods online approach with a convenience sample of Black and African American scientists to identify barriers to NIH grant review participation. Eligible participants were recruited online from professional organizations with primarily Black and African American membership. From February through April 2021, participants were asked to identify barriers to serving on NIH SRGs using concept mapping. Participants brainstormed statements describing barriers to serving on NIH SRGs, sorted statements into content themes, and rated statements on how true they were. Multidimensional scaling and a hierarchical cluster analysis identified content themes. Data analysis was conducted in May and June of 2021.

**Main Outcomes and Measures:**

Self-reported barriers to serving on an NIH SRG among Black and African American scientists.

**Results:**

A total of 52 scientists participated in both phases of the study (mean [SD] age, 42.3 [8.2] years; 46 women [88.5%]). Participants provided 68 unique statements that were organized into 9 thematic clusters describing barriers to serving on NIH SRGs. Themes included structural racism, diversity not valued, toxic environment, review workload demand, lack of reward, negative affect about the review process, competing demands at home institution, lack of opportunity, and perceptions of being unqualified.

**Conclusions and Relevance:**

Black and African American scientists reported many barriers to serving on NIH SRGs that are unique to Black and African American scientists, as well as barriers that transcend race but are exacerbated by structural racism. This study provides NIH with concrete opportunities to address realized barriers to increase inclusion of Black and African American scientists on NIH SRGs, fund more Black and African American scientists, and ultimately reduce health inequities in the US.

## Key Points

**Question:**

What are the perceived barriers that prevent Black and African American scientists from serving on National Institutes of Health (NIH) scientific review groups?

**Findings:**

This qualitative study of 52 scientists identifying as Black and/or African American develops descriptive themes for barriers that may prevent Black scientists from serving as NIH grant reviewers. Themes developed included structural racism, diversity not valued, toxic environment, review workload demand, lack of reward, negative affect about the review process, competing demands at home institution, lack of opportunity, and perceptions of being unqualified.

**Meaning:**

These results suggest that Black and African American scientists perceive undue barriers that prevent participation in the NIH grant review process; these barriers should be addressed to promote health equity.

## Teaser

This qualitative study identifies and thematically synthesizes barriers associated with lack of Black representation on National Institutes of Health (NIH) scientific review groups.

## Introduction

The National Institutes of Health (NIH) rely on reviews provided by members of scientific review groups (SRGs) to inform which grant applications submitted to NIH will be funded. Scientists are invited to serve as reviewers on SRGs, and while there are no concrete criteria, the Center for Scientific Review has described general requirements for candidates to be selected to serve as reviewers on SRGs. These criteria include being recognized as an authority in one’s field as evidenced by history of extramural funding, publication history, professional status and/or record of accomplishments, and past experience reviewing grants.^1^ One general requirement listed by the Center for Scientific Review indicates there must be “diversity with respect to geographic distribution, gender, race, and ethnicity of the membership.”^1^ However, data describing the characteristics of past SRG members demonstrate the lack of diversity with respect to race.

In 2011, *Science* published results of a detailed analysis by Ginther et al^2^ of US National Institutes of Health (NIH) investigator-initiated R01 awards from fiscal years 2000 through 2006. Results demonstrated systemic bias against Black and African American investigators, whose applications were 10% less likely to be funded relative to their White counterparts. This difference was statistically significant and negatively impacted Black and African American applicants’ career trajectory and the likelihood of successfully achieving the NIH goal, “to exemplify and promote the highest level of scientific integrity, public accountability, and social responsibility in the conduct of science.”^3^ A 2019 follow-up report^4^ analyzing NIH funding rates from 2011 to 2015 demonstrated that significant systemic racial bias remains, and identified 3 factors that contribute to it: (1) applicant research topic choice, (2) SRG decision-making processes to discuss applications, and (3) individual SRG reviewer priority score assignment. All 3 of these factors arguably reflect on the NIH review process. When considering the role SRGs play in sustaining systemic racial bias in NIH R01 funding, one fact is inescapable: while 77.8% of SRG members were White between 2011 and 2015, only 2.4% were Black. This abysmally low rate of Black and African American inclusion on NIH SRGs supports a negative feedback loop. That is, Black scientists are more likely than White scientists to submit grant applications on topics related to disparities in treatment and health outcomes based on race.^4^ With fewer Black scientists who have expertise in health disparities serving as SRG members, applications from Black researchers are less likely to be valued and funded. Fewer funded projects led by Black scientists leads to fewer Black reviewers serving on SRGs, as NIH grant funding history is considered in reviewer selection.^1^ In this way, inadequate Black and African American participation in NIH SRGs perpetuates low funding rates for Black scientists. These embedded racial inequities persist, are unacceptable, and call for intentional change.

Structural racism is defined as “the totality of ways in which societies foster racial discrimination through mutually reinforcing systems of housing, education, employment, earnings, benefits, credit, media, health care, and criminal justice. These patterns and practices in turn reinforce discriminatory beliefs, values, and distribution of resources.”^5^ In March 2021, NIH Director Dr Francis Collins announced the launch of UNITE, an NIH initiative “to end structural racism and racial inequities in biomedical research”^6^ that included a committee tasked with performing “a broad systematic evaluation of NIH extramural policies and processes to identify and change practices and structures that perpetuate a lack of inclusivity and diversity within the extramural research ecosystem.”^7^ Among other necessary steps that must be taken to end NIH’s history of structural racism, several groups have called for the elimination of underrepresentation of Black reviewers in NIH SRGs.^8,9^ Likewise, scholars have called for deeper understanding of the underpinnings of the NIH grant review process that sidelines Black scientists.^2^ The ability to end structural racism at NIH will depend upon learning from and acting upon firsthand reports of the lived experience of Black and African American scientists. In particular, efforts to reverse decades of Black and African American underrepresentation on SRGs likely will be most successful if NIH leadership is informed by and works to address barriers identified by Black and African American scientists that prevent them from participating in the NIH review process. To address this issue, we conducted a qualitative study to examine Black and African American scientists’ perceived barriers to NIH SRG participation.

## Methods

### Procedures

We sought to identify scientists (PhD, MD, or equivalent) who self-identified as African American and/or Black and would be actively involved in health-related research and likely actively involved in NIH grant writing. We used concept mapping^10^ to describe barriers identified by a convenience sample of scientists. Participants were recruited from research- and health-focused professional organizations, societies, and networks whose membership is primarily Black scientists in February 2021. Specifically, an email describing the study purpose and inviting Black and African American scientists to complete a screening questionnaire was sent to health and research society or organization announcement listservs (with administrator approval), including the American Public Health Association, the Association of Black Psychologists, College on Problems of Drug Dependence, National Black Nurses Association, National Medical Association, Society for Behavioral Medicine, and Society for Research on Nicotine and Tobacco. Recruitment emails invited those who identified as African American and/or Black scientists to participate in the study. At the study website, participants were asked to identify from a list of options which best described their race and ethnicity. All participants selected Black or African American. Ninety-nine individuals responded to the recruitment emails and completed the screening questionnaire. Email invitations to participate in the study were sent to those who completed the online screener and 52 provided consent (with a response rate of 52.5%).

The study was determined to be exempt by the East Carolina University and Medical Center institutional review board. A waiver of documentation of consent was obtained and participants provided consent at the study website (The Concept Systems; Global MAX) and completed an online questionnaire requesting demographic and relevant professional characteristics (eg, grants received, publications, grant review experience).

### Concept Mapping Methods

In a brainstorming task, participants generated statements that completed the following prompt: “A specific barrier, reason, challenge, or something else that has prevented me or would prevent me from agreeing to serve as an NIH grant reviewer is….” Participants entered statements at the study website that completed the prompt and described barriers preventing them from serving as an NIH grant reviewer. Participants were encouraged to enter multiple statements; statements were recorded in an ongoing list at the study website such that participants could view statements provided by previous participants. The brainstorming task ended when additional participants did not yield unique content. Participants generated 96 statements and each received $10 for brainstorming.

Statements were reviewed for redundancy and nonresponsiveness by 3 PhD scientists (S.F., a Black woman; R.N., a Black man; and T.E., a White man). If 2 or 3 reviewers indicated that multiple statements were redundant, the simplest or most representative was retained, leaving 68 statements. In March 2021 (approximately 1 month after the brainstorming task), participants who completed the brainstorming task were invited back to the study website to complete the sorting and rating tasks. Participants organized statements into themes by sorting statements into groups of similar content at the study website. After sorting, participants rated each statement on the prompt, “This is a barrier that I have experienced or felt that either has or would prevent me from agreeing to serve as an NIH grant reviewer,” using a 7-point scale (with 1 representing “Definitely NOT true for me,” and 7 “Definitely true for me”). Thirty-eight (73.1%) of the participants who completed brainstorming returned to the study website and completed sorting and rating (and received $35), consistent with recommended sample sizes required to complete data analysis.^11^

### Data Analysis and Representation

Nonmetric multidimensional scaling was used to create a map where each statement was represented by a single point. Each point’s location was assigned using an algorithm^12^: points that were closer together represented statements that were sorted together by more participants and thus represented similar content. The stress of the model (ie, fit indicator ranging from 0 to 1 that indicates congruency between the raw sorting data and the multidimensional scaling analysis) was 0.18, similar to previous work and indicated good model fit.^10^ Next, a hierarchical cluster analysis was conducted to create a cluster map by using an algorithm^13^ to identify clusters of statements by limiting the distance between the points and the centroid of nonoverlapping polygons, starting with a 2-cluster model. Subsequent models were examined by using statistical software to separate 1 cluster from the previous model into 2 new clusters (ie, models were created hierarchically from previous models). This procedure assigned statements into clusters based on how the participants grouped them in the sorting task. Multiple models were examined to determine the best fitting model through group discussion using parsimony (fewest clusters preferred) and interpretability (statements within a cluster describe a single idea or theme) as indicators of good fit until a final model was reached.

## Results

### Participant Characteristics

Participants were mostly women (46 participants [88.5%]); the mean (SD) age was 42.3 (8.2) years (Table 1). Two-thirds were tenured or tenure track (34 participants [65.4%]) with a rank of Assistant or Associate Professor (37 participants [71.1%]). Over half (43 participants [57.7%]) had been NIH grant principal investigators and 20 (38.5%) had served on an NIH SRG.

**Table 1.** Sample Characteristics

Characteristic	Participants, No. (%) (N = 52)
Age, mean (SD), y	42.3 (8.2)
Gender	
Women	46 (88.5)
Men	6 (11.5)
Ethnicity	
Hispanic/Latino(a)	1 (1.9)
Race	
Black/African American	52 (100.0)
Highest degree	
Doctorate degree (eg, PhD, DrPH, ScD)	48 (92.3)
Clinical or professional degree (eg, MD)	4 (7.7)
Current position	
Tenure track faculty—not tenured	19 (36.5)
Tenured faculty	15 (28.9)
Fixed term or non–tenure track faculty	7 (13.5)
Researcher not affiliated with a university	5 (9.6)
Other	6 (11.5)
Current rank^a^	
Postdoctoral fellow/researcher	4 (7.7)
Research scientist	3 (5.8)
Assistant professor	19 (36.5)
Associate professor	18 (34.6)
Full professor	5 (9.6)
Other	3 (5.8)
Field of research	
Social and behavioral sciences	11 (21.2)
Basic sciences	1 (1.9)
Health sciences	7 (13.5)
Public health	23 (44.2)
Medicine	2 (3.9)
Engineering	2 (3.9)
Other	6 (11.5)
Previous experience as principal investigator on NIH grant^b^	
F mechanism	4 (5.8)
R01 mechanism	11 (15.9)
Non-R01 R mechanism (eg, R03, R15, R21, R35, etc)	14 (20.3)
K mechanism	14 (20.3)
Other NIH grant	4 (5.8)
None	22 (31.9)
Previously asked to be an NIH SRG reviewer	20 (38.5)
Previously served as an NIH SRG reviewer	20 (38.5)

Abbreviations: NIH, National Institutes of Health; SRG, scientific review group.

^a^ All academic ranks include the equivalent positions in other (eg, international) systems.

^b^ Respondents could choose multiple options.

### Concept Mapping Results

The best fitting model included 9 thematic clusters (Figure and Table 2). The highest rated clusters were described under the categories structural racism, diversity not valued, and toxic environment. Structural racism (mean [SD] rating, 4.66 [0.67]) described how applications submitted by Black and African American scientists are reviewed more critically than those submitted by White scientists, SRGs are comprised of few Black and African American reviewers, senior White researchers dismiss reviews by Black and African American scientists, and senior White researchers are part of a “good old boy” network of researchers. Statements also described not wanting to participate in a process that discriminates against Black and African American scientists and that NIH does not appear interested in fixing the problem. Diversity not valued (mean [SD] rating, 4.62 [0.68]) described perceptions of prioritizing applications submitted by investigators from White elite institutions and/or who were regular grant recipients. Statements also described perceptions that NIH and SRG members lacked interest in social challenges, disparities, or justice-centered health equity research. Other statements suggested that NIH did not seek connections with Black and African American scientists and engaged in tokenism. Toxic environment (mean [SD] rating, 4.43 [0.13]) described navigating an adversarial environment and fatigue from working to feel respected.

**Figure.**
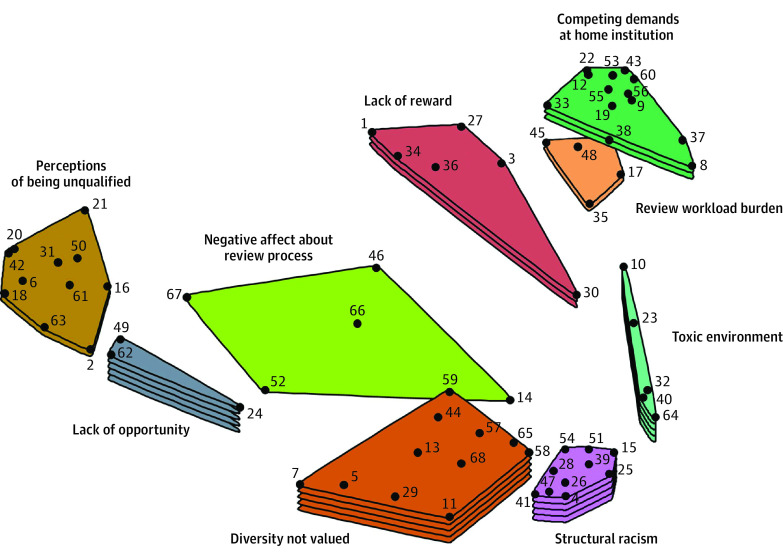
Concept Map of Barriers to Serving on a National Institutes of Health Scientific Review Group Points on the map represent statements generated in the brainstorming task. Numbered points correspond to the numbered statements listed in Table 2. Points closer to one another represent statements that were sorted together by more participants in the sorting task and thus represent more similar content. Clusters with greater number of layers represent clusters that had higher mean ratings of statements within the cluster.

**Table 2.** Clusters of Statements Describing Barriers for Black and African American Scientists Serving on National Institutes of Health (NIH) Scientific Review Groups

Statement^a^	Mean (SD) rating^b^
**Structural racism**	
Overall	4.66 (0.67)
54. The NIH priorities are behind in terms of awarding critical research that improves the lives of Black people and other minoritized peoples.	5.61
51. It is disheartening to always be the only Black person in a room full of scientists.	5.26
15. It is frustrating to see many White people study us—they are the PIs and the Black people are the workers.	5.26
28. The NIH is interested in surface level changes but not structural changes to create an environment that Black scientists would like to participate in.	5.18
26. White reviewers often make statements that acknowledge that they are part of the “good-old-boy” network and you are not, like they know people I do not know.	4.74
4. It is discouraging to see blatant differences in reviews, with grants submitted by Black PIs being reviewed more critically than those submitted by White PIs.	4.53
39. More senior White reviewers automatically side with their friends as opposed to trying to better understand a differing perspective.	4.50
25. I do not want to be a part of a racist structure that discriminates against Black scientists.	4.16
47. Bias on study sections where more senior White reviewers are often dismissive of my review.	4.03
41. Black reviewers sometimes do not consider unique perspectives of African American culture in efforts to seem inclusive of everyone and not give the impression that Black scientists receive special consideration.	3.29
**Diversity not valued**	
Overall	4.62 (0.68)
5. The NIH grant review process seems to be skewed toward persons who are regular recipients of R01 grants.	5.97
58. There is a prioritization of persons stemming from educational backgrounds that are reflective of elite Ivy League institutions, which are inherently White.	5.34
29. Many reviewers are not knowledgeable about community-engaged research and/or cultural factors unique to the location studied, but they become the experts focusing on the “methods.”	5.05
7. Overemphasis of reviews on genetics and biology rather than social challenges and disparities.	5.00
44. I believe access to opportunities to review is political—if you are not liked by the right people they may not facilitate this process.	4.79
57. There does not appear to be much interest among NIH staff in connecting with young researchers of color.	4.58
65. NIH does not really want my scientific input, but rather my physical presence.	4.50
59. Many researchers of color have had to opt for careers outside of academia due to the inability to receive NIH research grants to launch labs.	4.13
11. The NIH does not truly value the research I do, specifically justice-centered health equity research.	4.05
68. White administrators have not supported me in serving as an NIH grant reviewer because I was not funded by NIH. They say they want Black scientists to succeed but it does not feel that way.	3.71
13. There are very few women who are NIH grant reviewers.	3.66
**Toxic environment**	
Overall	4.43 (0.13)
32. Fatigue from always having to fight to get my point of view across, valued, or respected.	4.59
40. Mental fatigue in reading infuriating language that posits racial disparities as a product of biological factors rather than social factors.	4.55
64. Applications addressing African Americans seem to focus on getting funded rather than wanting to help advance the science/population.	4.45
10. I do not want to navigate the politics of a study section.	4.29
23. There is a cut-throat environment.	4.28
Review workload burden	
Overall	3.73 (0.83)
48. The number of proposals to review is excessive.	4.55
17. The long time spent at a study section meeting with few breaks is not representative of best practices in human health.	4.24
35. It is discouraging to put so much work into reviewing so many grants when only 2-3 out of your study section will get funded.	4.05
38. The amount of travel required is a lot.	3.63
45. I am asked to review often on multiple study sections during the same review cycle.	2.18
**Competing demands at home institution**	
Overall	4.15 (0.98)
9. A significant time commitment is required when serving on an NIH study section.	5.47
55. I need to prioritize getting publications.	5.21
60. The timing of requests to review and my competing demands.	4.82
37. I have a lot on my plate already as an early career person of color.	4.82
19. It is difficult to serve on NIH study section and work on writing my own grants.	4.76
8. I have limited capacity to review due to extensive service work and Black tax issues, such as diversity, equity, and inclusion initiatives.	4.42
43. I am overengaged in service activities at my institution which makes it difficult to take on additional reviews.	4.39
22. I do not receive release time to do NIH reviews.	3.95
56. My position/appointment does not allow time specifically for reviewing grants or serving on a study section.	3.73
33. I have limited capacity for research due to institutional support issues.	3.14
53. My teaching work at my institution is extensive and I sometimes just don’t have the energy to do a lot more.	3.05
12. Clinical duties can be difficult to rearrange in order to serve when study section meets.	1.95
**Lack of reward**	
Overall	3.98 (0.53)
27. The honorarium for reviewing is very low.	4.58
1. The task seems daunting.	4.55
34. There is no clear reward structure for participating that I am aware of.	4.14
3. There is very little reward.	4.03
30. The decision to remove continuous submission as a benefit of substantial ad hoc service.	3.38
36. Serving as an NIH grant reviewer sounds boring.	3.18
**Perceptions of being unqualified**	
Overall	3.71 (0.70)
61. Lack of mentorship on the initial process of becoming an NIH reviewer.	5.11
63. I am not known well enough by my research to be asked to review.	4.32
6. Being unaware of helpful programs like the Early Career Reviewer or CURE programs.	4.21
20. I am not sure how to become a reviewer.	3.87
42. I have little information about the NIH grant review process.	3.79
50. Lacking confidence that I am qualified to review grants.	3.79
16. I have not been awarded an NIH grant.	3.76
18. I am not sure if I have the required expertise to serve as a reviewer.	3.34
21. I have little information about how it relates to my career trajectory.	3.18
31. I am not senior enough to serve on a study section.	3.14
2. The study section that wanted my service was not in my area of expertise.	2.29
**Lack of opportunity**	
Overall	4.43 (0.54)
49. I have never been invited to serve as an NIH grant reviewer.	4.89
62. NIH does not ask junior investigators without an NIH grant to review.	4.74
24. Lack of training in this area due to preferential emphasis on advisors mentoring White trainees.	3.68
**Negative affect about the review process**	
Overall	3.41 (0.65)
66. I feel it is too risky to critique others because everyone else knows each other—it is hard to put myself out there.	4.03
67. I would feel very intimidated being on a panel with many well-funded reviewers.	4.00
52. I feel my ability to contribute to a less discriminatory review process is minimal as a more junior scientist.	3.55
14. I am too discouraged by the treatment of my own proposals to be fair to others.	3.18

Abbreviation: PI, primary investigator.

^a^ Statement numbers correspond to statement numbers displayed in the Figure.

^b^ Mean cluster ratings are calculated by averaging the mean rating for each statement within a cluster across all participants who completed the rating task.

Other themes were review workload burden, competing demands at home institution, and lack of reward. Review workload burden (mean [SD] rating, 3.73 [0.83]) described extensive travel, many applications assigned for review, and discouragement due to few funded applications. Competing demands at home institution (mean [SD] rating, 4.15 [0.98]) described unique Black and African American faculty demands, including what could be referred to as a “Black tax” (ie, disproportionately greater service), serving on diversity, equity, and inclusion initiatives, and mentoring students of color. Statements described having positions with extensive teaching and/or service requirements and no grant reviewing time release. Statements described how the time commitment was made more challenging due to publishing scientific manuscripts and seeking grant funding. Lack of reward (mean [SD] rating, 3.98 [0.53]) described how being an NIH reviewer was daunting with limited incentives.

Two clusters described perceptions of being unqualified and lack of opportunity. Perceptions of being unqualified (mean [SD] rating, 3.71 [0.70]) described lack of mentorship or knowledge about becoming an NIH reviewer, not having a strong scholarly reputation, not having been awarded an NIH grant, or lacking confidence in possessing the required expertise. Lack of opportunity (mean [SD] rating, 4.44 [0.54]; range, 3.68-4.89) described not being asked to review, perceiving that NIH does not invite junior investigators without NIH grants, and preferential mentoring of White trainees. The central cluster, negative affect about the review process (mean [SD] rating, 3.41 [0.65]), described apprehension about critiquing applicants who are known by reviewers, feeling intimidated in the presence of well-funded reviewers, and limited ability to contribute to a less discriminatory process due to lack of seniority.

## Discussion

Our study documented the perceived barriers that prevent Black and African American scientists from serving as NIH SRG members. Black and African American scientists report facing many barriers that may prevent them from serving as an NIH SRG member. The most strongly endorsed barrier was the perceived structural bias by the NIH and other SRG members. Structural bias permeated several clusters, including lack of diversity on SRGs and SRG toxic environment. Some identified barriers transcend race; however, these barriers are exacerbated for Black and African American scientists as evidenced by statements describing a “Black tax,” limited training or mentorship opportunities due to preferential support of White mentees, and navigating toxic environments that epitomize structural racism in academia. The UNITE initiative represents progress toward ending the pattern of structural racism at NIH. Current actions include “understanding stakeholder experiences,” providing resources for “new research on health disparities, minority health, and health equity,” “improving the NIH culture and structure for equity,” ensuring transparency in the extramural funding research process, and “changing policy, culture and structure to promote workforce diversity.”^7^ These broad initiatives and goals may be informed by the results of the current study, with specific actions that could be taken outlined below.

Certainly, NIH can make purposeful efforts to invite more Black and African American scientists to participate in SRGs, and accountability for these efforts could be improved by reporting details about the process annually. However, inviting additional Black and African American scientists to serve as NIH reviewers in the absence of additional actions that address the perceived barriers that prevent Black and African American scientists from accepting invitations will not address the issues outlined in the current study. The themes identified by the Black and African American scientists who participated in this concept mapping study provide addressable areas to improve the NIH SRG process. For example, to address barriers identified in the perceptions of being unqualified cluster, NIH could take actions to increase Black and African American scientists’ experience and confidence in reviewing, such as providing meaningful and funded training and mentoring experiences to Black and African American scientists to support SRG participation. Because statements indicated that Black and African American scientists may not be supported by their home institutions to participate in grant reviews, funding mechanisms could be established that would provide financial support to allow Black scientists to serve on SRGs and be compensated during their time of service.

Given that systemic bias on NIH SRGs was identified as an important barrier, NIH could increase efforts to assess and intervene against biased actions of scientists and SRG chairs participating in NIH SRGs. The NIH Center for Scientific Review can be charged with conducting ongoing SRG review and providing comprehensive training to identify and prevent biased behavior. Additionally, NIH could invite more reviewers with expertise in social determinants related to health disparities and social justice to ensure that applications focusing on health equity and social justice are reviewed equitably. This approach would be consistent with the Center’s identified need that review groups examining multidisciplinary applications have scientist participants “who have broader expertise or who have demonstrated the capacity to appreciate and evaluate areas of science outside their immediate area of expertise.”^1^ Therefore, increasing Black and African American representation on SRGs will likely result in greater appreciation of applications that focus on health disparities and social determinants of health.

### Limitations

This study had several limitations. The sample was a nonprobability sample of Black and African American scientists. The vast majority of participants were women and most were from social and behavioral sciences or public health disciplines. Additionally, participants were recruited primarily from professional society or organization email listservs. Therefore, the barriers identified may be less generalizable to other Black scientists, such as men or transgender scientists, those in basic science or clinical fields, or Black and African American scientists who are not members of the societies and organizations that were included in recruitment. However, Black women account for approximately two-thirds of the degrees conferred upon Black doctoral students^14^ and therefore our sample was representative of the scientific environment. The study included 52 participants: this number of participants is sufficient and consistent with other concept mapping studies used for strategic planning.^11^ Future studies with larger samples and with greater representation of Black and African American scientists may be able to identify additional barriers to serving on SRGs.

## Conclusions

Our findings can inform programming like UNITE and aid NIH’s efforts to reduce racially biased barriers to increase Black participation. With the UNITE initiative, NIH has an opportunity and obligation to reduce racially biased barriers to NIH SRG diversity and inclusion. This effort must address participation of Black reviewers in NIH SRGs and unbiased consideration of applications for research projects on topics related to social determinants affecting health outcomes, which are often submitted by Black and African American scientists. With unbiased reviews, the probability of successful funding of Black and African American scientist could increase Black membership on NIH SRGs. Likewise, providing a better reward structure for SRG members, providing opportunities for NIH grant reviewer mentorship, cultivating an environment free of structural racism, and welcoming social justice focused research will likely increase Black and African American representation on SRGs. Intervening to increase Black and African American scientist representation on NIH SRGs will lead to greater funding of Black scientists and ultimately contribute to greater health equity.
